# Predictors of hyporesponsiveness to ESAs in peritoneal dialysis patients: the role of residual renal function

**DOI:** 10.1590/2175-8239-JBN-2022-0019en

**Published:** 2022-07-25

**Authors:** Marisa Roldão, Hernâni Gonçalves, Francisco Ferrer

**Affiliations:** 1 Centro Hospitalar do Médio Tejo, Serviço de Nefrologia, Torres Novas, Portugal

Dear Editor,

Anemia resistant to recombinant human erythropoietin (rhEPO) is a risk factor for mortality in patients with chronic kidney disease (CKD) on dialysis^1^. Several factors such as uremic toxins, iron deficiency, inflammation, hyperparathyroidism, and medications have been associated with EPO hyporesponsiveness in hemodialysis (HD) patients, but evidence on peritoneal dialysis (PD) is limited^2^. Identifying causes of hyporesponsiveness may help to overcome the resistance in these patients.

We conducted a cross-sectional study involving 50 prevalent PD patients. Patients with acute or chronic inflammatory disease, active malignancy and those not receiving rhEPO therapy were excluded. Patients were treated with weekly darbepoetin alfa to maintain hemoglobin (Hgb) concentration between 10-12 g/dL. To evaluate the dose-response effect of rhEPO therapy, we used the erythropoietin resistance index (ERI), calculated as the mean weekly weight-adjusted dose of rhEPO (U/Kg per week) divided by the mean hemoglobin level (g/dL), over a 3-month period. EPO hyporesponsiveness was defined as an ERI value above the upper quartile (>10), so patients were classified in two groups: ERI≤10 and ERI>10. We compared clinical, analytical, and demographic data among groups. Body composition and fluid volume were evaluated by bioimpedance using the body composition monitor (BCM). Logistic regression analysis was performed to identify predictors of rhEPO hyporesponsiveness. Statistical analysis was executed using SPSS. The mean age of 50 prevalent PD patients was 52.04 ± 15.98 years, 29 (58%) were male, 29 (58%) were diabetic, and 31 (64%) were treated with angiotensin-converting enzyme inhibitors (ACEi) or angiotensin receptor blockers (ARB). Mean hemoglobin level (Hb) was 10.99 ± 0.81 g/dL and mean ERI was 7.64 ± 7.25. Eleven patients (22%) had hyporesponsiveness to rhEPO therapy (ERI>10). There was no difference among groups for age, gender, etiology of chronic kidney disease, or PD modality. There was also no difference in the use of ACEIs or ARBs. Hyporesponsive patients had lower body mass index (BMI) (22.94 ± 2.89 vs 26.74±4.53 Kg/m^2^, *p*=0.01) and lower lean tissue index (LTI) (9.96 ± 1.94 vs 16.23 ± 18.51 Kg/m^2^, *p*=0.02), but similar fat tissue index (FTI). Weekly creatinine clearance (peritoneal plus urinary), but not Kt/V, was also significantly lower in this group (68.76 ± 37.29 vs 87.84 ± 35.35 mL/min/1.73m^2^, *p*=0.028). Hyporesponsive patients had lower urine volume (0.73 ± 0.63 vs 1.39 ± 0.67 L, *p*=0.005) and residual kidney function (RKF) (3.43 ± 3.04 vs 6.13 ± 3.69 mL/min/1.73m^2^, *p*=0.044). The proportion of patients with fluid overload, defined as overhydration (OH)/extracellular water (ECW) >15%, was significantly higher in this group (*p*=0.04). No difference between groups was observed in albumin, c-reactive protein, intact parathormone, serum ferritin, or transferrin saturation index. Using a logistic regression analysis, we observed that BMI [(OR) 0.56 (CI: 0.364-0.849)] and LTI [(OR) 0.315 (CI: 0.130-0.767)] were predictors of hyporesponsiveness to rhEPO therapy in an unadjusted model.

In our cohort, body composition, fluid status, and RKF were the main factors that affected the response to rhEPO therapy. In addition to the similarity of our results to those previously described by Penne et al. concerning a strong relationship between RKF and improved phosphate and anemia control in HD patients^3^, our study also shows the importance of euvolemia in the response to rhEPO. The mechanisms underlying these findings are not clear, but we could postulate that they are related to the “inflammatory state” of CKD patients. In our population, factors previously associated with EPO resistance, such as iron status, did not differ significantly between groups. However, these results should be interpreted with caution, since a wide variance in ferritin and TSAT was found in those classified as “hyporesponders”.

Once again, our results support the importance of RKF-preserving strategies and euvolemia maintenance in prevalent PD patients, not only to improve patient and technique survival but also to overcome rhEPO resistance.

**Table 1:** Clinical and analytical sata of PD patients classified according to ERI groups

	ERI ≤ 10	ERI > 10	*p*-value
Patients, *n* (%)	39 (78%)	11 (22%)	
Age (years) mean ± SD	54.15 ± 15.73	44.55 ± 15.25	0.078
Male, *n* (%)	23 (59%)	6 (54.5%)	0.793
ESKD etiology, *n* (%)			0.126
Diabetes mellitus	12 (30.8%)	3 (27%)	
Hypertension	10 (25.6%)	3 (27%)	
Cardiorenal syndrome	4 (10.3%)	1 (9%)	
Unknown	4 (10.3%)	1 (9%)	
Other	9 (23%)	3 (27%)	
Comorbidities, *n* (%)			
Diabetes mellitus	23 (59%)	6 (54.5%)	0.793
Hypertension	27 (69.2%)	6 (54.5%)	0.364
ACEIs/ARBs, *n* (%)	26 (66.7%)	6 (54.5%)	0.459
PD modality, *n* (%)			
APD	29 (74.36%)	8 (72.73)	0.962
**Urine Output (mL)** mean ± SD	**1339.5 ± 667.3**	**726.5 ± 628.8**	**0.006**
**Residual Kidney Function (mL/min/1.73m^2^)** mean ± SD	**6.13 ± 3.69**	**3.43 ± 3.04**	**0.039**
**BMI (kg/m^2^)** mean ± SD	**26.74 ± 4.53**	**26.74 ± 4.53**	**0.012**
**LTI (kg/m^2^)** mean ± SD	**16.23 ± 18.51**	**9.96 ± 1.94**	**0.020**
FTI (Kg/m^2^) mean ± SD	12.31 ± 6.21	10.6 ± 2.3	0.194
**OH/ECW ≥15%, *n* (%)** mean ± SD	**5 ± 13.9**	**4 ± 44.4**	**0.040**
**Hgb (g/dL)** mean ± SD	**11.21 ± 0.66**	**10.21 ± 0.87**	**0.001**
Ferritin (ng/mL) median (IQR)	154 (170.9)	233 (287.4)	0.280
TSAT (%) median (IQR)	25 (23)	29 (18)	0.527
Albumin (g/dL) median (IQR)	3.2 (0.76)	3.43 (1.38)	0.504
CRP (mg/dL) median (IQR)	0.5 (0.8)	0.5 (0.7)	0.532
Ca^2+^ (mg/dL) mean ± SD	8.55 ± 0.7	8.45 ± 0.4	0.220
P^+^ (mg/dL) mean ± SD	4.9 ± 0.96	5.29 ± 1.25	0.269
iPTH (ng/mL) median (IQR)	332.7 (332)	132 (384)	0.406
Weekly Kt/V	2.36 ± 0.53	2.72 ± 0.69	0.517
**Weekly CrCl (mL/min/1.73m^2^)**	**87.84 ± 35.35**	**68.76 ± 37.29**	**0.028**

*P*-values from Student’s T-test for normally distributed continuous variables, Mann-Whitney U-test for continuous variables with skewed distributed, and Chi-square test for categorical variables. ESKD: end-stage kidney disease; CRP: c-reactive protein; BMI: body mass index; LTI: lean tissue index; FTI: fat tissue index; Hgb: hemoglobin; TSAT: transferrin saturation; ACEIs: angiotensin-converting enzyme inhibitors; ARBs: angiotensin receptor blockers: Ca^2+^: calcium; P+: phosphorus; iPTH: intact parathormone; CrCL: creatinine clearance; IQR: interquartile range.
